# The Dental and Oral Science Magazine

**Published:** 1878-06

**Authors:** 


*The Dental and Oral Science Magazine.*—Notice of this new Dental Journal was made in our April number. We are glad to see this new competitor for position. It appears handsomely and if, as the prospectus states, it is to publish the proceedings of the New York Odontological Society *in full*, it certainly will not lack for matter—though we are puzzled to see how Mr. Williams, the publisher, will get all of this Society's voluminous utterances into the compass of the seventy-two pages of a quarterly. He will have to use smaller type than at present, and leave out his selections.

The announcement is made in the prospectus that "trade in all its phases will be rigorously excluded from its literary department, in which no puffs or advertisements of any business, any material, or any individual, will be allowed to appear." And it further states that "it is designed to give the new periodical as high a character for scientific professional literature as can be found in the journals of this or any other profession; and to obtain this end, much must necessarily be excluded which, possessing merely local or temporary value, makes no mark on the record of professional excellence."

It is certainly difficult to understand how any fact in oral or dental surgery could possess "temporary or local value," but it may be that New York is cosmopolitan in this, as in trade; or is it that the New York Odontological Society is the oracle of the profession?

Besides the triangular "New Departure," by Drs. Flagg, Chase and Palmer, this number of the quarterly contains a very thoughtful and interesting article from Dr. Norman W. Kingsley, on "The Mechanism of Speech," and an excellent paper by Dr. C. A. Marvin, of Brooklyn, on "The Relation of the Science of Physiognomy to Mechanical Dentistry," a study which dentists might well profit by, for the good of their patients. But so long as artificial dentistry is assigned to boys in the laboratory, it is not likely that progress will be made, but rather retrogression.

